# The national burden of frailty and disproportionate distribution of its components—the predominance of slow gait speed: a 2018–19 face-to-face epidemiologic assessment representative of population of older Poles

**DOI:** 10.1007/s40520-022-02331-5

**Published:** 2023-01-12

**Authors:** Karolina Piotrowicz, Hanna Kujawska-Danecka, Kacper Jagiełło, Adam Hajduk, Anna Skalska, Małgorzata Mossakowska, Tomasz Zdrojewski, Tomasz Grodzicki, Jerzy Gąsowski

**Affiliations:** 1 Department of Internal Medicine and Gerontology, Faculty of Medicine, Jagiellonian University Medical College, 2 Jakubowskiego St., 30-688, Kraków, Poland; 2 Department of Internal Medicine, Connective Tissue Diseases and Geriatrics, Medical University of Gdansk, Gdansk, Poland; 3 Department of Preventive Medicine and Education, Medical University of Gdańsk, Gdansk, Poland; 4 Study On Ageing and Longevity, International Institute of Molecular and Cell Biology, Warsaw, Poland

**Keywords:** Pre-frailty, Frailty, Frailty components, Population study, Gait speed

## Abstract

**Background:**

The prevalence of frailty and its components may be affected by age, diseases and geriatric deficits. However, the current operational definition of frailty assigns equal weight to the five components of frailty.

**Aims:**

To perform a population-based assessment of physical frailty, its prevalence, and distribution of its components across different age, disease and deficit spectrum.

**Methods:**

From 2018 to 2019, we conducted a face-to-face cross-sectional assessment of a representative sample of older Poles. We obtained data on frailty components, chronic disease burden, and prevalence of particular diseases and geriatric deficits. We calculated weighted population estimates, representative of 8.5 million older Poles, of prevalence of frailty and its components across the disease burden, associated with the particular diseases and the geriatric deficits present.

**Results:**

Of 10,635 screened persons ≥ 60 years, 5987 entered the face-to-face assessment. Data of 5410 have been used for the present analysis. Seventy-two percent of the population are burdened with at least one frailty component. The estimated weighted population prevalence (95% CI) of frailty was 15.9% (14.6–17.1%), and of pre-frailty 55.8% (53.3–58.2%). Slow gait speed predominated across disease burden, specific diseases, geriatric deficits and the age spectrum. Overall, the prevalence of slow gait speed was 56.3% (52.7–60.0%), followed by weakness 26.9% (25.4–28.4%), exhaustion 19.2% (17.6–20.8%), low physical activity 16.5% (14.8–18.3%), and weight loss 9.4% (8.4–10.3%).

**Conclusions:**

Slow gait speed predominates among the components of frailty in older Poles. This may affect the component-tailored preventive and therapeutic actions to tackle frailty.

## Introduction

Linda Fried et al. introduced the operational definition of physical frailty syndrome, a phenotype that includes weakness, slow gait speed, exhaustion, weight loss and physical inactivity [1]. When untreated, physical frailty has been linked to disability, falls and fractures, increased morbidity, and mortality [2, 3]. Additionally, greater load of chronic conditions also leads to greater disability, partly through direct means, but additionally by promoting physical frailty [2]. The unfortunate price we pay for longer lives is more years living with disability [4]. One way to prevent disability in older persons is to fight frailty and its components.

The operational definition of frailty assigns equal weight to each of the components where the fulfillment of each of the criteria yields a score of 1 [1]. However, the prevalence of the particular components may differ between populations, across the chronic disease burden, in particular diseases, and in particular geriatric deficits. This means that different weights for particular components of frailty might be required to increase the performative value of the scale. Indeed, the data obtained from Far-Eastern populations indicate that frailty components may be distributed unevenly [5, 6]. However, similar data are lacking for the majority of Western populations. Further, it is currently unknown how the particular components of frailty associate with increasing age, diseases and geriatric deficits, and whether the prevalent pattern of components seen among persons with pre-frailty and frailty changes with accretion of chronic conditions and multimorbidity. We set out to describe, based on the results of a recent, country-wide representative study of aging in Poland, the frequency of frailty syndrome components across the disease and the geriatric deficit categories. We hypothesize that the five components of frailty should be approximately equally distributed across the different categorizations.

## Methods

### Study design and oversight

The PolSenior2 project was a nationwide, multicenter, cross-sectional, face-to-face study of health and its determinants in old age conducted between 2018 and 2019 in Poland [7]. A random, three-stage, proportional sampling procedure, stratified by age and sex, was employed to select a study group of 5987 Polish community-dwelling adults ≥ 60 years of age, representative of the general population of older Poles, estimated at 8.5 million. Exclusion criteria consisted of inability to establish contact with a selected respondent, hospitalization or institutionalization, other temporary relocations, death prior to the beginning of the study, refusal to participate, inability to obtain informed consent. The study selection and procedures were described in detail and published elsewhere [7]. The PolSenior2 project was approved by the Bioethics Committee of the Medical University of Gdańsk, Poland (NKBBN/257/2017). Written consent was obtained from all participants prior to enrollment in the study.

### Study procedures

Frailty syndrome was diagnosed based on Fried et al.’s physical frailty phenotype criteria [1]. It is comprised of weakness, slow gait speed, self-reported exhaustion, low level of physical activity, and unintentional weight loss [1]. Muscle strength was checked three times in both hands with a handheld hydraulic dynamometer (Saehan SH5001) in a standard seated position [8]. Weakness was defined based on the sex and body build stratified cut-off values for hand grip strength as established by Fried et al. [1]. Time to walk a distance of 3 m at usual pace was measured; if impossible because of home environment or safety conditions, a shorter walking distance was used. Slow gait speed was diagnosed in accordance with the walking time cut-offs, stratified by sex and height [1]. Exhaustion was diagnosed based on a positive response to at least one of the two modified questions of the Center for Epidemiologic Studies Depression Scale (CES-D): “I felt that everything I did in the last week was an effort” and “I could not get going” [9]. The 7-day Physical Activity Recall (PAR) scale was used to collect information on the type and duration of the subject’s physical activity in the last 7 days before the examination [10]. Based on the PAR, the individual weekly energy expenditure was estimated and classified according to Fried et al.’s low level of physical activity thresholds (with < 383 kcal/week and < 270 kcal/week for men and women, respectively) [1]. We recorded weight (in kilograms) using the Tanita BC-545 N Segmental Body Composition Scale and unintentional body weight loss (shrinkage) was diagnosed if a loss of > 4.5 kg or ≥ 5% in the past 6 months was confirmed. Obesity was defined as a BMI (kg/m^2^) ≥ 30. In respondents who met three or more physical frailty phenotype criteria, frailty syndrome was diagnosed; in those with one or two, pre-frailty was recognized [1]. Information on subjects’ comorbidities (min–max: 0–30) was gathered during home interviews, and included history of hypertension, arrythmias with atrial fibrillation, heart failure, past hospitalization due to coronary artery disease, stroke, epilepsy, Parkinson’s disease, psychiatric diseases with depression, pulmonary diseases (including chronic obstructive pulmonary disease, asthma, emphysema, chronic bronchitis, pulmonary fibrosis), chronic kidney disease, urolithiasis, history of recurrent or chronic urinary tract infections, peptic ulcers, cirrhosis and hepatitis B or C infection, malignancies and hematologic diseases, diabetes, dyslipidemias, thyroid and ophthalmic diseases (glaucoma, cataracts, age-related macular degeneration), and hearing impairment. Additionally, subjects’ medications, blood and urine laboratory results, and blood pressure measurements were considered for diagnosis of the following: hypertension (SBP ≥ 140 or DBP ≥ 90 mmHg or on antihypertensive medications), diabetes (fasting glucose > 125 mg/dl or on antidiabetic medications), chronic kidney disease (estimated glomerular filtration rate (eGRF) < 60 ml/min/1.73 m2 or albumin-to-creatinine ratio (ACR) ≥ 30 mg/g) and thyroid disease (TSH > 4.78 mIU/l or TSH < 0.1 mIU/l or L-thyroxin therapy or thyrostatic therapy). Participants were screened for cognitive impairment with the Mini-Mental State Examination (MMSE), and classified as suspected dementia when scoring < 24 points. In those with MMSE score of at least 19 points, the 15-item Geriatric Depression Score (GDS) to screen for depressiveness was used. We suspected depression if the subject reported a depressed mood or scored > 5 points in the GDS. Hearing impairment was noted if a hearing problem was noted during the interview. Visual acuity was tested with Snellen charts. The European Working Group on Sarcopenia in Older People revised consensus (EWGSOP2) was employed for the diagnosis of probable sarcopenia [8], malnutrition and its risk was diagnosed based on the MNA-SF scale (< 12 points). We obtained information concerning stress and urge incontinence, a 12-month history of falls, symptoms of anorexia of aging (past 3 months), pain (duration of at least 3 months, severity of at least 4 out of 10), level of independence in activities of daily living (ADL; ADL-based disability in those with ADL < 5 points).

### Statistical analyses

The data management and the statistical analyses were performed with R version 3.6.3 R (R Core Team, version 3.6.3) and SAS 9.4 TS Level 1M5 (SAS Institute Inc., Cary, NC, USA). The continuous variables were compared with t-tests or Mann–Whitney U tests for two groups and in the case of three or more groups, ANOVA or Kruskal–Wallis tests were used for normally and non-normally distributed variables, respectively. The proportions were compared with chi-square test. Sampling weights were included in statistical calculations to account for the complex survey design using R survey package. The post-stratification procedure was used to match age–sex sample distribution to the population of Poland. The two-tailed tests were carried out with significance level of *p* ≤ 0.05.

## Results

**Fig. 1:**
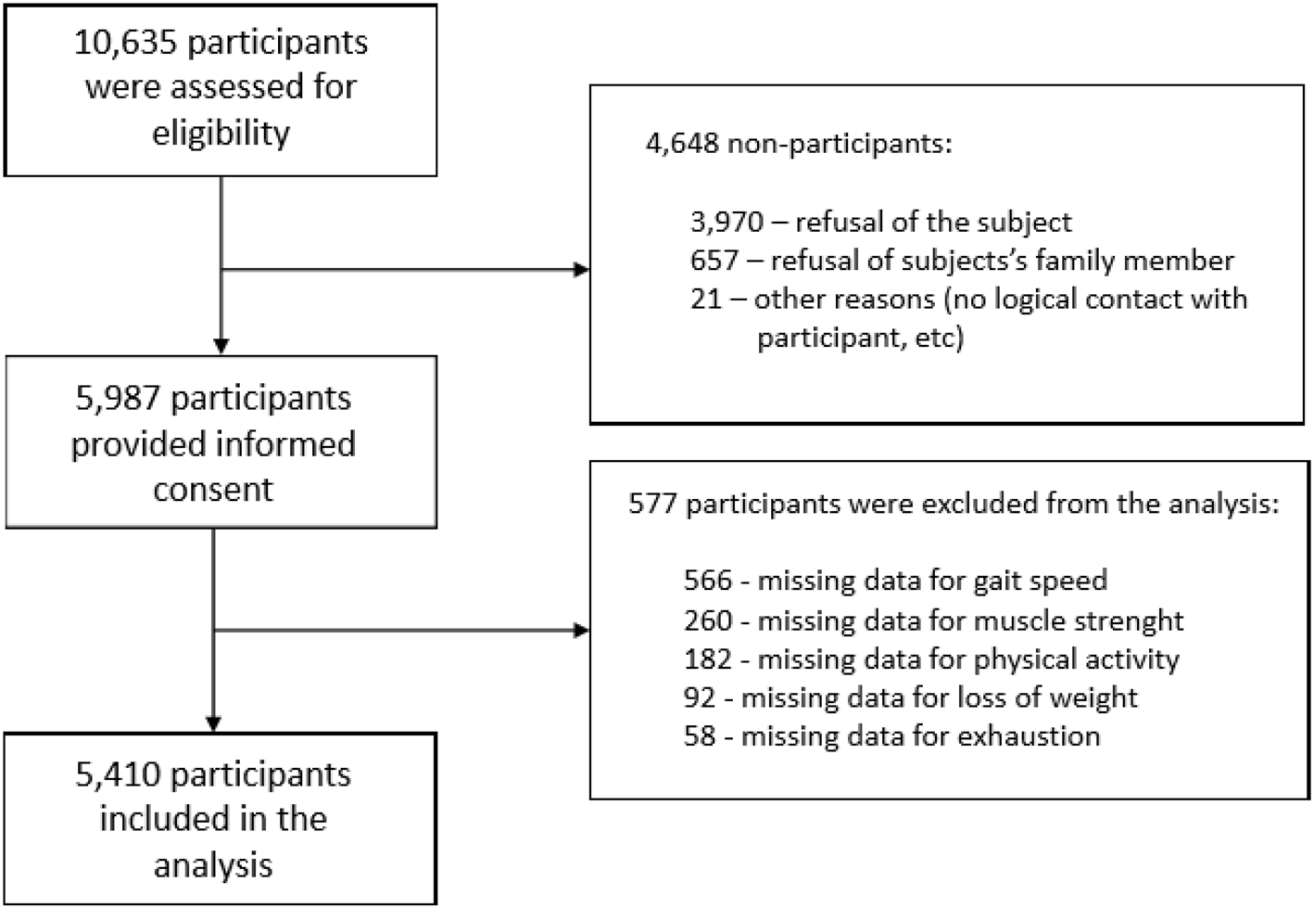
The flow of the participants

Of the 10,635 screened persons, 5,987 (56%) were included in the face-to-face assessment phase of the study. Presently, we show the data of 5,410 (50.6% women) participants of the PolSenior2 survey for whom we had the complete data concerning frailty syndrome components, frequency of diseases, and geriatric deficits (Fig. 1). The mean (standard deviation, SD) age was 75.0 (9.5) years. The percentage of participants in the four age-cohorts was: 34.1% 60–69 years, 32.6% 70–79 years, 24.6% 80–89 years, and 8.7% 90 + years.

**Fig. 2:**
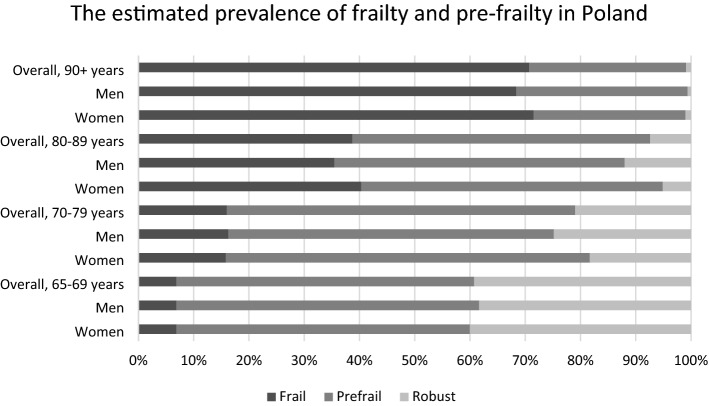
The estimated prevalence of frailty and pre-frailty in Poland

The characteristics of the studied group are contained in Table 1. Overall, the estimated weighted population prevalence (95% CI) of frailty was 15.9% (14.6–17.1%), and that of pre-frailty 55.8% (53.3–58.2%). Estimated prevalence of pre-frailty did not differ between men and women. Frailty was more frequent among women than men [17.4% (15.8–19.0) vs. 13.8% (12.2–15.3%)]. The prevalence of both pre-frailty and frailty, both in men and women, increased across the age strata (Fig. 2). Primary education vs. all other forms of education has been associated with the highest prevalence of frailty (Table 1). Among robust persons, we estimated the percentage of persons burdened with 0, 1, 2, and 3 + diseases at 9.5% (6.3–12.7%), 15.8% (12.6–19.1%), 22.3% (19.1–25.5%), 52.4% (47.9–56.9%), respectively. The corresponding prevalence among the pre-frail persons was 6.4 (4.5–8.3%), 14.4% (12.5–16.3%), 16.9% (14.7–19.0%), 62.4% (59.2–65.6%). Among the frail persons, the estimated frequencies averaged 2.3% (1.0–3.6%), 6.4% (4.7–8.1%), 10.4% (7.4–13.4%), 80.9% (76.8–84.9%), for 0, 1, 2, 3 + diseases, respectively. Among the frail and the pre-frail persons, the composite of cardiovascular diseases were the most frequent, reaching 81.9%, and 76.6%, respectively. Among the most frequent disorders and impairments prevalent in more than 50% of frail patients were depression (58.0%), visual impairment (62.8%), pain (57.8%), incontinence (57.8%), malnutrition (70.9%), and sarcopenia (79.0%).

**Table 1:** The characteristics of the population

Estimated prevalence or estimated mean value of	Women	Men	p	Robust	Pre-frail	Frail	*p*
Age	71.5 (71.3–71.6)	69.6 (69.5–69.8)	< 0.001	66.7 (66.4–66.9)	70.7 (70.4–71.0)	78.0 (77.3–78.7)	< 0.001
Education			< 0.001				< 0.001
Primary	28.5 (24.5–32.6)	19.6 (16.8–22.4)		12.0 (9.1–14.9)	25.7 (21.7–29.7)	44.8 (39.2–50.4)	
Vocational	20.2 (17.4–22.9)	36.3 (33.5–39)		28.7 (24.5–33)	27.8 (25.1–30.5)	20.7 (16.9–24.5)	
Secondary	37.4 (34.3–40.6)	30.3 (27.5–33)		39.3 (35.4–43.2)	34.2 (31.4–37)	26.4 (21.9–30.9)	
Higher	13.8 (10.6–17.1)	13.9 (11.1–16.7)		20.0 (15.5–24.4)	12.3 (9.8–14.8)	8.1 (5.5–10.7)	
Residence			0.090				0.120
Rural	37.5 (30.2–44.9)	39.5 (33.1–45.8)		35.8 (27–44.6)	38.8 (31.9–45.6)	41.2 (33.2–49.2)	
Small town (< 50 thousand)	22.3 (15.9–28.8)	21.1 (15.8–26.3)		25.2 (15.9–34.6)	20.6 (15.6–25.7)	19.8 (14.0–25.6)	
Medium-sized town (50–200 thousand)	16.0 (11.1–21)	18.4 (13.7–23.1)		14.2 (8.5–20)	19.0 (14–24)	15.1 (10.3–19.9)	
Cities > 200 thousand	24.1 (11.7–36.5)	21.1 (11.2–31)		24.7 (11.4–38)	21.6 (11–32.2)	23.9 (12.5–35.3)	
Occupation			< 0.001				< 0.001
Blue collar	41.8 (37–46.6)	60.5 (57–63.9)		44.1 (39.4–48.8)	50.7 (46.4–55)	57.2 (52.2–62.1)	
White collar	42.0 (36.7–47.3)	21.6 (18.9–24.3)		38.8 (33.6–43.9)	32.5 (28.4–36.5)	25.8 (20.7–30.8)	
Farmer	7.6 (5.2–9.9)	5.8 (3.3–8.2)		4.0 (1.8–6.2)	7.4 (4.8–10.1)	9.9 (6.7–13.2)	
Services	6.7 (5.5–7.9)	10.5 (8.4–12.7)		10.8 (8.1–13.4)	7.7 (6.4–9.1)	5.8 (3.7–7.9)	
Marital status			< 0.001				< 0.001
Bachelor	3.5 (2.7–4.3)	3.7 (2.6–4.9)		3.8 (2.1–5.5)	3.1 (2.3–3.8)	5.1 (2.9–7.2)	
Married	51.2 (48.8–53.6)	82.0 (79.9–84)		75.4 (72.5–78.3)	63.8 (61.8–65.9)	44.5 (40.5–48.4)	
Widow/Widower	39.8 (37.7–41.9)	10.7 (9.2–12.3)		15.4 (13.2–17.6)	28.5 (26.4–30.5)	47.1 (43.2–50.9)	
Divorced/Separated	5.5 (4–7)	3.6 (2.5–4.6)		5.4 (3.2–7.6)	4.7 (3.6–5.7)	3.4 (1.7–5)	
Smoking	11.4 (9.6–13.2)	18.0 (15.4–20.6)	< 0.001	17.5 (13.6–21.3)	13.6 (11.8–15.3)	10.5 (7.2–13.8)	
Number of frailty components (%)			0.007				
0	26.5 (23.5–29.5)	31.0 (27.6–34.3)		100.0	0	0	
1	37.5 (34.7–40.3)	37.2 (34.3–40)		0	67.0 (64.5–69.5)	0	
2	18.6 (16.6–20.6)	18.1 (15.8–20.4)		0	33.0 (30.5–35.5)	0	
3	11.5 (10.3–12.7)	9.2 (8–10.4)		0	0	66.5 (63.7–69.4)	
4	5.4 (4.5–6.3)	3.8 (3.1–4.6)		0	0	30.0 (27.3–32.7)	
5	0.5 (0.2–0.7)	0.7 (0.4–1)		0	0	3.5 (2.3–4.6)	
Number of diseases			< 0.001				< 0.001
0	5.6 (3.7–7.5)	8.2 (6.4–9.9)		9.5 (6.3–12.7)	6.4 (4.5–8.3)	2.3 (1.0–3.6)	
1	11.6 (9.9–13.2)	16.5 (14.1–18.9)		15.8 (12.6–19.1)	14.4 (12.5–16.3)	6.4 (4.7–8.1)	
2	16.4 (14.5–18.3)	18.9 (16.5–21.3)		22.3 (19.1–25.5)	16.9 (14.7–19)	10.4 (7.4–13.4)	
3 or more	66.4 (63.3–69.6)	56.5 (53.4–59.5)		52.4 (47.9–56.9)	62.4 (59.2–65.6)	80.9 (76.8–84.9)

**Fig. 3:**
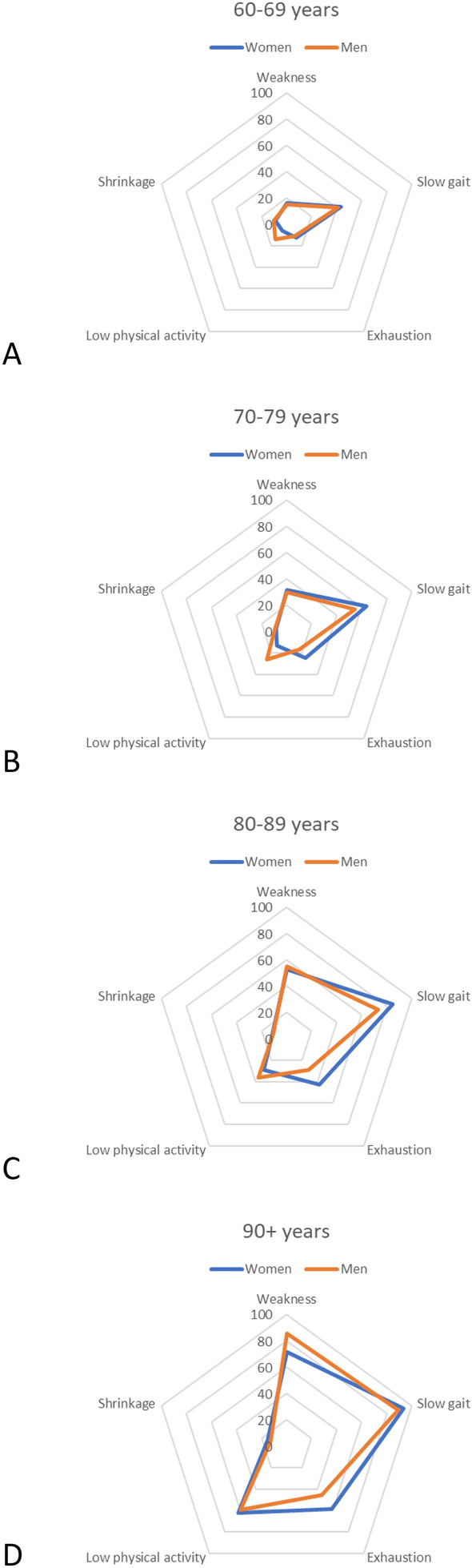
Components of frailty according to sex and age, the entire population

**Fig. 4:**
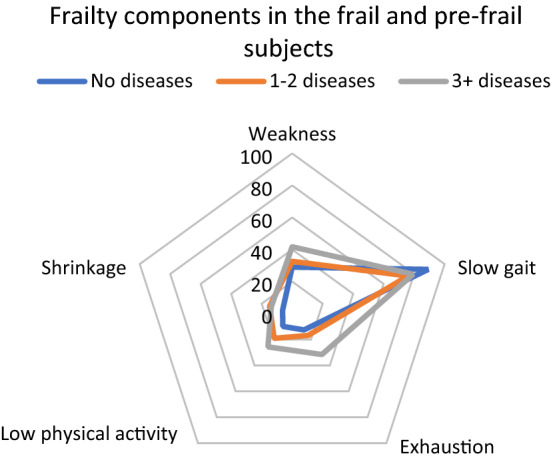
Components of frailty according to the disease burden. Pre-frail and frail persons

The overall population estimates of the prevalence of particular frailty components were as follows: 56.3% (52.7–60.0%) had slow gait speed, 26.9% (25.4–28.4%) had weakness, 19.2% (17.6–20.8%) had exhaustion, 16.5% (14.8 –18.3%) had low physical activity, and 9.4% (8.4–10.3%) had weight loss. Across the age strata, both in men and women, slow gait speed predominated (Fig. 3). The pattern in which slow gait speed predominated was consistent across the 0, 1–2, 3 + disease strata (Fig. 4).

In Tables 2 and 3, we present the estimated (with 95% CI) prevalence of particular frailty components in persons burdened with particular diseases (Table 2) or geriatric deficits (Table 3).

**Table 2:** Frequency of particular frailty components among persons with given disease. Estimates for Poland

(%, 95%CI)	All cardiovascular	Heart failure	All pulmonary disease	COPD	Stroke	Parkinson’s	CKD	Ophthalmologic pathology	Cancer	Peptic ulcer disease	DM	Thyroid disease	Obesity
Slow gait speed	58.6 (55.0–62.3)	68.6 (64.0–73.3)	58.3 (51.5–65.2)	71.4 (61.7–81.1)	71.1 (64.4–77.8)	89.7 (82.8–96.6)	73.7 (70.0–77.4)	67.3 (63.2–71.4)	59.0 (50.3–67.7)	53.5 (47.9–59.1)	64.2 (59.8–68.7)	56.0 (51.2–60.8)	58.6 (54.3–62.9)
Weakness	29.2 (27.3–31.1)	41.5 (37.4–45.7)	31.9 (27.8–36.0)	39.0 (31.0–47.0)	45.7 (38.7–52.7)	56.8 (42.1–71.5)	46.9 (43.3–50.5)	27.9 (25.0–30.7)	29.1 (24.7–33.4)	25.0 (21.4–28.7)	34.2 (30.8–37.6)	24.7 (21.7–27.8)	32.3 (29.7–34.9)
Exhaustion	20.5 (18.7–22.3)	30.7 (26.8–34.6)	26.7 (22.4–31.0)	33.0 (26.2–39.8)	39.8 (33.3–46.3)	50.2 (36.6–63.7)	32.9 (29.1–36.6)	35.7 (32.8–38.6)	22.2 (18.0–26.4)	22.1 (18.5–25.7)	25.6 (22.5–28.8)	22.2 (19.4–25.0)	22.1 (19.4–24.8)
Low physical activity	17.9 (15.9–19.9)	25.8 (22.1–29.5)	21.3 (17.4–25.2)	30.8 (21.8–39.8)	34.5 (27.8–41.1)	35.9 (23.9–47.9)	30.6 (26.9–34.3)	21.9 (19.0–24.9)	19.8 (15.6–24.0)	15.7 (12.6–18.9)	23.0 (19.8–26.3)	13.6 (10.8–16.3)	16.5 (14.1–19.0)
Shrinkage	9.5 (8.4–10.7)	11.7 (9.0–14.3)	13.0 (9.9–16.0)	12.4 (7.6–17.2)	9.4 (6.5–12.3)	10.6 (1.9–19.3)	11.6 (9.6–13.5)	10.9 (9.2–12.6)	9.8 (7.1–12.5)	12.9 (9.8–16.1)	12.3 (10.2–14.5)	10.0 (8.0–12.1)	8.5 (6.9–10.1)

**Table 3:** Frequency of particular frailty components among persons with given geriatric deficit. Estimates for Poland

(%, 95%CI)	Mood disorders	Cognitive impairment	Visual impairment	Hearing impairment	Pain	Incontinence	Anorexia of aging	Malnutrition	Sarcopenia	Falls	ADL < 5 points
Slow gait speed	71.9 (67.3–76.5)	70.2 (64.9–75.4)	67.3 (63.1–71.5)	79.3 (73.9–84.8)	61.1 (57.1–65.2)	64.1 (59.7–68.5)	72.5 (67.7–77.4)	74.6 (71.0–78.3)	79.3 (75.7–83.0)	72.9 (68.1–77.6)	97.1 (93.5–100.0)
Exhaustion	41.8 (38.2–45.4)	39.8 (35.1–44.6)	27.0 (24.6–29.4)	45.8 (41.1–50.4)	28.4 (25.5–31.4)	28.5 (26.0–31.0)	39.0 (34.3–43.7)	43.4 (40.0–46.8)	31.4 (28.7–34.2)	39.0 (34.2–43.9)	77.7 (71.4–83.9)
Weakness	39.4 (36.1–42.8)	42.2 (37.9–46.4)	32.5 (30.0–35.1)	53.3 (47.4–59.1)	30.6 (28.3–32.9)	35.6 (32.5–38.6)	39.5 (34.8–44.2)	41.4 (38.4–44.3)	38.9 (36.2–41.6)	41.4 (37.1–45.8)	71.0 (62.1–79.9)
Low physical activity	24.7 (20.8–28.6)	31.5 (27.4–35.5)	22.3 (19.6–25.0)	36.3 (31.7–41.0)	16.9 (14.5–19.4)	19.5 (17.3–21.8)	25.9 (21.6–30.2)	30.6 (27.3–33.9)	25.8 (23.3–28.3)	27.7 (23.5–31.8)	66.5 (59.7–73.4)
Shrinkage	12.0 (9.7–14.2)	11.3 (9.0–13.7)	10.4 (8.9–12.0)	8.4 (5.7–11.1)	10.3 (8.7–12.0)	10.1 (8.4–11.8)	26.8 (22.6–31.1)	29.8 (26.6–33.0)	9.4 (8.1–10.8)	12.3 (9.5–15.1)	11.4 (7.1–15.7)

Slow gait speed predominated across increasing number of diseases (48.5% in persons with one disease to 59.1% in persons with 3 + diseases), geriatric deficits (61.1% in persons with chronic pain to 79.3% in persons with sarcopenia and 73.9% in persons with hearing impairment), and loss of functionality (97.1% in persons with ADL < 5) (Table 2, 3).

The estimated prevalence of slow gait speed ranged between particular diseases from 53.5 (47.9–59.1) for peptic ulcer disease to 89.7 (82.8–96.6) for Parkinson’s disease (Table 2).

From lowest to highest age strata, the percentage of other components, predominantly weakness, exhaustion, and in the oldest persons, low physical activity, grew, reaching the highest values in the cohort of 90 + years (Fig. 3).

## Discussion

We demonstrated that in the sample representative of the total population of 8.5 million older Poles, of all frailty components it is slow gait speed that predominates irrespective of age, number of chronic diseases, presence of particular diseases, and geriatric deficits. Our data, containing the population estimates based on a face-to-face assessment of the representative sample of older Poles, present the last available pre-COVID-19 picture of the problem of frailty which is of epidemic proportions. This is important both at the individual and the societal level. Among the population of 8,547,800 persons at or above the age of 60 years currently living in Poland [11], we show that an estimated 1,358,000 (1,251,000–1,466,000) persons are burdened with frailty and 4,775,000 (4,558,000–4,992,000) are pre-frail.

The population estimates of frailty differ between the published reports. The data from the sixth wave of the Survey of Health, Ageing and Retirement in Europe (SHARE) show that the prevalence of frailty in selected European countries ranges from 3.0% in Switzerland to 15.6% in Portugal. The SHARE estimate of frailty prevalence for Poland is 13.1% [12]. However, their group contained a large proportion of individuals aged between 50 and 59 years. In contrast, we give the first national comprehensive estimates of frailty in persons at or above the age of 60 years. Additionally, the methods used to draw the sample allow weighing within age strata; thus, our sample is representative in each age stratum. Putting our results in a broader context, the analysis of the Health and Retirement Study (HRS; 2004–2012) showed that the estimated prevalence of frailty in the older US population was 12.4% [13]. Based on the data from the Chinese Longitudinal Healthy Longevity Study (CLHLS, 2008–2018), in the population aged 65 years or more, with a mean age of 85.8 years, the prevalence of frailty was 26.3%. In frail persons exhaustion (83.2%), slow gait speed (83%) and weakness (82.5%) predominated [14].

In our cross-sectional analysis, we demonstrated that slow gait speed was the most prevalent frailty component. This was true across sex, age, and disease burden strata. In the oldest older adults, weakness, low physical activity, and to some extent, exhaustion were also increasing in prevalence. Among the pre-frail and frail persons combined, the pattern in which slow gait speed predominated was constant across the disease burden strata.

In frail persons from CLHLS, weakness was associated with 62%, slow gait speed with 67%, and inactivity with 61% greater risk of death. This was in stark contrast with the mortality risk associated with exhaustion (18%), and shrinkage (11%) [14].

Despite the fact that CLHLS data tend to indicate that in older persons frailty comes first and augments the risk for development or clinical manifestation of other disorders [14], further prospective studies are needed to tackle the problem which of the either and in whom comes first.

Several recent studies reported the over-representation of slow gait [15], the prognostic significance of slow gait speed in conditions ranging from cancer to liver dysfunction [16, 17], and cardiovascular mortality [18, 19]. However, no comprehensive analysis based on national-level representative data that would cover the broad range of conditions, geriatric deficits and age range has been published thus far.

For the first time, in a nationwide representative population face-to-face assessment study, we demonstrate that the components based on which physical frailty can be diagnosed are not uniformly present in the older echelons of the society. The predominance of slow gait speed may point to the fact that society may be largely sedentary. The data from The Irish Longitudinal Study on Ageing (TILDA), indicate a trend towards increasing prevalence of slow gait speed (9.8, 15.3, 13.5, 20.3, 24.9%) and low physical activity (14.4, 14.2, 19.5, 32.0, 33.7%) over the follow-up of participants between 2009 and 2018 [20]. In line with TILDA, based on our cross-sectional data, we found that older persons tended to have higher prevalences of slow gait speed and low physical activity. However, the possibility exists that the increased prevalence of slow gait speed and low physical activity is not only the effect of individual aging but also of the possible societal tendency towards a more sedentary lifestyle. This may have become even more important during the COVID-19 pandemic [21]. Our finding that slow gait speed is the most prevalent component of frailty in older Poles has potentially far-reaching implications.

The importance of gait speed as a geriatric measure has been established. It has been estimated that 0.1–0.2 m/s slower gait translates into 11% greater risk of 30-day mortality in cardiovascular surgery [22, 23].

In our study, slow gait speed is followed, in terms of frequency, by weakness. When considering single components of physical frailty, persons with exhaustion tended to have a higher prevalence of geriatric deficits. We found that the ADL-based diagnosis of disability was more prevalent in persons with exhaustion and low physical activity. The FRéLE study demonstrated that low physical activity and slowness were related to both physical and cognitive aspects of basic and instrumental activities of daily living [24]. Slow gait speed both in frail and non-frail older individuals has been associated with cognitive decline [25], and has been associated with the development of social frailty [26, 27]. At the population level, frailty is expensive. Japanese data indicate that over the next 20 years, the estimated costs of frailty in Japan alone will reach $97 billion [28].

Our study needs to be considered in the context of its limitations. The study was a cross-sectional survey, hence no cause–effect considerations are possible. As an example, the neurologic deficits associated with previous stroke or ongoing Parkinsonism are associated with slow gait speed and weakness, and frailty at large. However, despite the obvious possible influence of stroke or extrapyramidal pathology on gait speed, it is likewise possible that frailty presents a sort of umbrella effect lensing the overall homeostatic disequilibrium that facilitates poor neurological outcomes [29]. The pathophysiology of slow gait speed may differ between the particular diseases and our study was not designed to offer mechanistic explanations of studied phenomena. The possibility exists that the culturally conditioned low level of physical activity rests at the pathophysiologic background of the high prevalence of pre-frailty and frailty. Likewise, it is translating into an epidemic of slower gait in the geriatric segment of the population. When accumulated beyond the cut-off for frailty, this yields a pathophysiologic background to, or shares a common pathophysiologic environment with, the preferential development or revelation of certain pathologies. When confirmed using more data, especially prospective data, this might point to effective population-level strategies to counter the burden of frailty and its aftermath.

In our study, the gait speed assessment was performed by a trained nurse in the participant’s home, and despite the same recommended distance, the standardization was difficult. Likewise, weight loss has been retrospectively assessed over the period of 6 months based on the questionnaire. This may have led to a bias in the assessment of shrinkage.

In conclusion, the population of older Poles is heavily burdened with pre-frailty and frailty. The fact that slow gait speed comes to the forefront of the particular components of physical frailty sets the stage for possible preventive and therapeutic measures. Knowing that certain diseases associate with particular components of frailty may serve as a warning during a comprehensive geriatric assessment. To elucidate the pathophysiologic and causative nature of the relations we describe, further prospective studies are needed.
